# Effectiveness of a question formulation rubric with second-year medical students: a randomized controlled trial

**DOI:** 10.5195/jmla.2023.1529

**Published:** 2023-04-21

**Authors:** Jonathan D. Eldredge, Melissa A. Schiff, Jens O. Langsjoen

**Affiliations:** 1 jeldredge@salud.unm.edu, Professor, Health Sciences Library and Informatics Center, Department of Family & Community Medicine, School of Medicine, University of New Mexico, Albuquerque, NM.; 2 mschiff@salud.unm.edu, Professor. Department of Internal Medicine, School of Medicine, University of New Mexico, Albuquerque, NM.; 3 jlangsjoen@salud.unm.edu, Associate Professor, Department of Internal Medicine, School of Medicine, University of New Mexico, Albuquerque, NM.

**Keywords:** Question Formulation, Evidence Based Practice, Scoring Rubrics, Medical Education, Educational Measurement

## Abstract

**Objective:**

The FAC (Focus, Amplify, Compose) rubric for assessing medical students' question formulation skills normally accompanies our Evidence Based Practice (EBP) training. The combined training and assessment rubric have improved student scores significantly. How much does the rubric itself contribute to improved student scores? This study sought to measure student improvement using the rubric either with or without a linked 25-minute training session.

**Methods:**

Randomized Controlled Trial. The authors tested the hypothesis that a 25-minute training session combined with use of a rubric would lead to higher scores than a brief explanation of this rubric alone. All 72 participating second-year medical students had a question formulation rubric briefly explained to them following a pre-test. Students in the intervention groups were taught how to formulate EBP questions for 25 minutes using the rubric followed with another 30 minutes of EBP search training. Students in the control group only received the 30 minutes of EBP search training in their small group labs. All 72 students took the post-test in which they formulated a question in response to a clinical vignette. Statistical analysis to test the hypothesis consisted of a two-sample paired t-test to measure between-group differences.

**Discussion:**

Both the intervention and control groups performed significantly better on the post-test for question formulation skills than on the pre-test. When analyzed by extent of individual improvement between pre- and post-tests using a two-sample paired t-test for between group differences, the control group students receiving only a brief explanation of the rubric performed the same statistically (intervention 37.7 versus 37.4 control) as the intervention group students who received the same brief explanation followed by a 25-minute active learning training session. Thus, the results provided no support of the hypothesis that the extra 25-minute training improved post-test scores. The rubric itself contributed similarly to the intervention groups students' improvement as the combined rubric and training for control group students. This finding could potentially save scarce curricular time.

**Key Messages:**

The FAC question formulation rubric and training significantly improves medical students' EBP question quality. The FAC rubric coupled with only a 5-minute explanation can be effective. In a crowded medical school curriculum, the rubric and brief explanation might save valued time for other purposes.

## INTRODUCTION

Evidence Based Practice (EBP) provides a durable, time-tested framework for making informed clinical decisions. EBP has been defined as “A way of providing health care that is guided by a thoughtful integration of the best available scientific knowledge with clinical expertise. This approach allows the practitioner to critically assess research data, clinical guidelines, and other information resources in order to correctly identify the clinical problem, apply the most high-quality intervention, and reevaluate the outcome for future improvement [1].” EBP usually consists of five steps: ask, access, appraise, apply, and assess. The first question formulation step (“Ask”) largely determines the effectiveness of the subsequent steps in the process, particularly the second step of searching for the evidence. A Cochrane Collaboration-sponsored systematic review on interventions to teach learners how to formulate questions underscored the importance of the topic, stating that, “Formulating questions is fundamental to the daily life of a healthcare worker [2].”

Almost all medical schools include training in the steps of the EBP process, although their approaches vary considerably [3–5]. EBP training begins in medical school and continues into medical residencies where residents are expected to achieve their EBP competencies [6–7]. Traditionally, many EBP instructors have employed the Patient, Intervention, Comparison, Outcome (PICO) approach to teaching question formulating skills. A group of physicians in 1995 invented the PICO question formulation approach [8]. Since then, the use of PICO has become ingrained in many EBP training sessions.

Upon reconsideration of the accumulating evidence, the PICO approach possibly has not proven itself to be as durable and applicable as the overall EBP framework. While initially incorporated into the canon of EBP training, it has come under increasing scrutiny. Huang et al. compared actual clinicians' questions with PICO and found PICO did not represent those questions well. Importantly, they also determined that PICO was most suitable *only for treatment* EBP questions rather than all of the other types of EBP questions [9]. Since only about half of the EBP questions relate to treatment [10–11] it seems likely that the PICO format does not adapt well to diagnosis, prognosis, epidemiology, or other types of EBP questions. Looking ahead to step two in the EBP process, Schardt et al. determined no statistical difference between either using PICO or not using PICO search templates for effective searching in PubMed [12]. Hoogendam et al. similarly found PICO to be deficient for launching a timed PubMed search[13]. A 2018 review of whether PICO improved the quality of searches in a variety of databases proved inconclusive [14]. No wonder then that Mintzler et al. recently depicted PICO as “an elephant in the evidence-based medicine classroom…”[15] Even health sciences librarians have reported problems with translating PICO formatted questions into effective and timely searches[16]. Efforts to correct the deficiencies with PICO sometimes have led to elaborate derivations. Davies' inventory of these derivations includes the acronyms ADAPTE, ECLIPSE, SPICE, PICOT, PICOTT, and PESICO. Most of these derivations relate more to librarians' work rather than to health care providers' or students' engagement with question formulation [17].

Eldredge et al. developed the Focus, Amplify, and Compose (FAC) system for formulating EBP questions. They had witnessed students struggling with adapting the PICO format to their own question formulation exercises. The FAC system was designed to position the formulated question to lead next to an effective search, the second step in the EBP process, and later the third critical appraisal step. A quasi-experiment conducted in 2019 revealed that medical students improved their question formulation skills using FAC by a statistically significant margin, thereby offering a plausible and more versatile alternative to PICO. The authors of this study speculated about the comparative utility of the rubric itself versus the rubric combined with training in teaching medical students how to in formulate effective EBP questions [18].

This present study conducted at the University of New Mexico School of Medicine (UNMSOM) built upon the previous quasi-experiment by comparing medical student performance in question formulation using the rubric with only a brief introduction versus student performance following a 25-minute active learning session using the same rubric. In a crowded curriculum [19]. with a premium on every contact hour, the authors conversely wondered if the EBP training with minimal explanation of the rubric could be streamlined to save time. The authors hypothesized that second-year medical students in an intervention Group who had 25-minutes of additional training and hands-on application exercises would outperform their control Group student counterparts who had only a brief introduction to the rubric.

## METHODS

**Figure 1:**
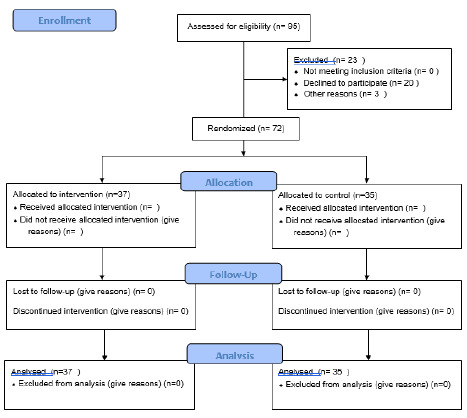
CONSORT Flow Diagram

Second year medical students (*n* = 95) were randomized into their small group assignments prior to the block. These small groups were randomized further using the Research Randomizer [20]. into either the control group or the intervention group in the BrightSpacetm learning management system prior to the beginning of the Quantitative Medicine Block that covers epidemiology, biostatistics, and evidence-based practice. Students are normally assigned to small group labs in their blocks so this was an expected routine. All medical students consent when they matriculate to be part of those research studies approved by the Institutional Review Board (IRB). The authors conducted the entire Quantitative Medicine Block online in Zoom. They leveraged the online Zoom platform during the Covid-19 Pandemic to minimize contamination between the two groups to conceal their activities from one another during the study. Control group students were assigned to the afternoon labs on December 7. Intervention group students were assigned to the afternoon December 8 labs. Figure 1 provides a flow diagram of the study. Table 1 describes the characteristics of the control and intervention group participants. The analysis of the participants on Table 1 indicates that the randomization worked correctly. The p-values measure the degree of difference between the groups statistically. Students could elect to participate or not participate in the ungraded formative exercises described in this study per an established UNMSOM policy.

**Table 1:** Characteristics of Participants

	Control	Intervention	P-Values
Elected to not participate *(n)*	10	10	—
Leave of Absence *(n)*	2	0	—
Female *(n)*	23	22	0.87
Male *(n)*	12	15	0.87
Final Exam Grade (%)	94.933	94.121	0.5908

On the first full day of the Quantitative Medicine Block, all 72 participating students present were given a one-hour introduction to Evidence-Based Practice (EBP). About ten minutes into the EBP introductory session the authors administered an ungraded, timed pre-test that the instructors referred to in a non-intimidating, matter-of-fact way in the BrightSpacetm learning management system to as a “Baseline Assessment” to gauge each student's individual skills level in formulating EBP questions. All medical students were already well acquainted with the BrightSpacetm learning management system. The students were given five minutes to complete the Baseline Assessment online prompted by a clinical vignette in the “Quizzes” segment of the “Activities” area of the BrightSpacetm learning management system.

On the first full day of the Quantitative Medicine Block, all 72 participating students present were given a one-hour introduction to Evidence-Based Practice (EBP). About ten minutes into the EBP introductory session the authors administered an ungraded, timed pre-test that the instructors referred to in a non-intimidating, matter-of-fact way in the BrightSpacetm learning management system to as a “Baseline Assessment” to gauge each student's individual skills level in formulating EBP questions. All medical students were already well acquainted with the BrightSpacetm learning management system. The students were given five minutes to complete the Baseline Assessment online prompted by a clinical vignette in the “Quizzes” segment of the “Activities” area of the BrightSpacetm learning management system:

You are at a rural clinic gaining practical experience. Today you are enjoying the work, although you miss your student friends back at the University of New Mexico School of Medicine. Manuel Garcia, age 73, is in the clinic. During the last two months Mr. Garcia's has experienced recurring leg tremors, complaints of “weakness,” apathy, slowness in his movements, unilateral rigidity, shuffling gait, and instability when walking. Your preceptor is seeing him today about Mr. Garcia's recent fall in his kitchen. Mr. Garcia appears to be fine, yet shaken from the fall. Your preceptor has diagnosed Mr. Garcia as having fairly advanced stage Parkinson Disease. You know about Parkinson Disease based on your courses at the University of New Mexico School of Medicine. The discussion expands to include possible drugs that might improve the quality of life for Mr. Garcia. Your preceptor discusses possibly prescribing Levodopa or a dopamine agonist.

Formulate a single-sentence question, based on this clinical vignette that, when answered by either you or other colleagues, will lead to the best treatment of this patient. Take no longer than five (5) minutes.

This is an ungraded exercise to help us evaluate your baseline skills so try your best.

Following a discussion of student observations on the challenges of formulating questions that they experienced during the pre-test exercise, the first author gave a five-minute overview of the FAC question formulation rubric (Table 2) to *all* students enrolled in the Quantitative Medicine Block. The first author walked the students through the sections of the FAC rubric, starting with the need to focus on the patient's central problem while removing any unnecessary information. Next, the Amplify section of the rubric offered prompts from the patient care context that might be included in the question. The Compose section of the FAC rubric pointed to the need to state the question in a single sentence that can stand alone. The development of the rubric has been described elsewhere [18]. Control group students, numbering 12-14 at a time, participated in their assigned hour-long labs at the designated time slots later the same day. They accessed their labs by assigned online Zoom links. Their lab attendance, as well as intervention group student attendance, was taken at the beginning and at the end of the lab sessions by a staff member unaffiliated with the study. The control group students received EBP search training during their labs. Instead of EBP question formulation training, these control group students were given additional time to practice their search skills.

**Table 2:** FAC Rubric for Evaluating Formulated EBP Questions

Element	Points
Focus	
Identifies and focuses upon the main problem or disease	15
Minimizes “noise” in formulated question by removing unneeded elements	5
Amplify the signal in the question, only if *applicable*, with:	
Descriptive Adjectives: (Examples: acute/chronic, insidious/abrupt, proximal/distal, sharp/dull)	5
Scale (Examples: neoplastic staging; child development Tanner stages)	2
Temporality (Examples: duration of illness; length of treatment; seasonality, etc.)	2
Describes the population aspects (age, geography, ethnicity, income)	6
Composition:	
Question accurately reflects contextual details	5
The final formulated question “stands by itself”	10
TOTAL POINTS (out of 50 possible points)	____/50

The intervention group students on December 8, 2020 received their EBP search skills training. They also accessed their labs by assigned online Zoom links. In contrast to the control group students, however, the intervention group students received a 25-minute practical, active learning training with the FAC question formulation rubric. The online session consisting of about 12-14 students per lab began with the opening solo thought question: “Why do you think that formulating answerable questions will be important for your individual professional education and for your career?” Students later offered their answers. The instructor (JE) described studies that revealed that practicing physicians, on average, raise questions at the rate of about one per every other patient. Using the FAC rubric the instructor walked through the elements of Focus, Amplify, and Compose. They were asked to analyze a sample question and determine the ways that it did or did not adhere to the elements of the rubric. The instructor displayed eight sample questions composed by medical students the preceding year to instill confidence in their own emerging skills. Students were presented with possible topic areas and asked to compose their own question using the rubric. They were then paired in a Zoom breakout room for 5 minutes to evaluate each other's question using the rubric. The instructor tried to visit each room, but usually made it to only two rooms before the five-minute period had expired. When they returned from their online breakout rooms, several students typically would share their refined questions and a few offered what they learned from the experience.

The next morning on December 9 all 72 participating students present were given a timed five-minute “Spot Check” (post-test) on their question formulation skills prompted by the same vignette, consistent with standard pre-test post-test [21] and rubric applications [22] with all students allowed to use the rubric. Neither the intervention nor the control groups received any training related to translating this vignette into an answerable EBP question. The students' pre- and post-tests for both the intervention and the control groups were administered within the “Quizzes” feature within the “Activities” area of the BrightSpacetm learning management system. All medical students were already well-acquainted with this learning management system so there were no technical issues or delays. Students' pre- and post-test formulated questions were transferred to Word documents for scoring. The identities of the students and their allocation to either the control or intervention groups were concealed from the faculty scorers (JE & MS) who used the same FAC rubric to score the students' EBP questions. The vignette provided all of the possible elements that could be used in the formulated question that then could be scored on the rubric.

Once all pre- and post-tests were scored, the data sets were transmitted to the statistician on an Excel spreadsheet with the identities of the intervention and control datasets removed in order to prevent the possible unconscious bias in her analysis. The scores generated during the pre-test compared to the post-test for the question formulation skills, and analyzed for differences between the intervention or control groups, are the basis of this study that received Institutional Review Board ethics approval (20-588) from the University of New Mexico Health Sciences Center on October 11, 2020.

## RESULTS

The intervention group average score was 13.1 out of a possible 50 points and the control group with 16.8 out of a possible 50 points on the pre-test as documented in Table 3. Comparing the intervention group to the control group, they did not differ on their pre-test scores (p value = 0.02675). Two of the authors have been on Curriculum Committee for several years so they knew that this cohort of students had not been exposed to any question formulation training as part of the curriculum so the authors expected an improvement from pre-test to post-test scores. Both the intervention and control groups performed significantly better on the post-test assessment scores than on their pre-test scores as analyzed in Table 3. This RCT post-test finding was consistent with previous quasi-experimental experiences involving the FAC rubric [18, 23].

**Table 3:** Results

	Intervention	Control	P-Values	Effect Size
Number	37	35		
Average Baseline Pre-Test Scores	13.1 (8.9 to 17.4)*	16.8 (11.7 to 21.9)*	0.2675	0.2654
Average Spot Check Post-Test Scores	37.7 (34.3 to 41.1)*	37.4 (33.1 to 41.7)*	0.9106	0.0263
Pre- to Post Differences	24.6 (19.2 to 30.0)*	20.6 (13.0 to 28.2)*	0.3849	0.2071
Range of Pre-Test Scores	40	46		
Range of Post-Test Scores	35	50		

* Ranges are confidence intervals at 95%confidence with an Alpha level set at 0.05

Table 3 indicates that neither the intervention nor the control groups differed significantly from one another on their post-test scores. Comparing the intervention to the control group, they did not differ on their post-test scores (p value = 0.9106) and their pre- to post-test differences (p value = 0.3849). When students were compared using a two-sample paired t-test for between group differences their degree of improvement between their individual pre- and post-test scores, the confidence intervals for differences between both the control group (CI = 13.0 to 28.2) and intervention group (CI = 19.2 to 30.0) post-scores overlap almost completely. Despite the hypothesis that the intervention group would have greater improvement in test scores, the results show no statistically significant differences between the two groups. These intergroup results were contrary to the hypothesis that the 25-minute session would improve post-test scores, thereby supporting the null hypothesis. At the same time, all students performed better on their post-tests compared to their pre-tests, thereby suggesting the power of the rubric when coupled to a five-minute explanation to guide second-year medical students to perform well on their assessments. In other words, the intervention group's 25-minute training session does not appear to have made a statistical difference in the post-tests from the control group's post-tests. The dataset can be accessed at the University of New Mexico's institutional repository.

## DISCUSSION

Both the intervention and control groups improved on their post-tests compared to their pre-test scores. At the same time, neither group significantly performed better statistically than the other as measured by post-test scores. Specifically, the intervention group's added 25-minute training did not lead to statistically better post-test scores compared to the control group. Medical educators often note that “Assessment drives the curriculum” and this adage might offer a partial explanation for these results that rejected the hypothesis. Our medical students are expected to take considerable responsibility for their own learning. From the block orientation onward, the students also knew that their EBP question formulation skills would constitute 5% of the final block grade on an assessment 12 days later in the block. One might infer that the students were motivated by this looming graded assignment. As for the lack of differences between the 25-minute intervention group and the solely 5-minute control groups experiences, the specific intervention training itself possibly was insufficient to improve students' skills more than the 5-minute alone explanation of the rubric. Although the three instructors have received multiple teaching awards for this Block, and their students have performed well on the Block topics on their national U.S. Medical Licensure Exam (USMLE) Step One, the extra 25-minute instruction segment still might have not been as effective as their instruction elsewhere in the Block so this factor must be considered among other potential limitations.

The present study confirmed that brief instruction and student use of a rubric led to significant improvement in post-test scores. Three previous randomized controlled trials involving health sciences students documented a similar overall improvement effect. A general pattern might be emerging from the evidence that library or informatics training leads to improvement. Yet, similarly, these **three** other studies also identified no statistical differences between intervention and control groups. Carroll et al. measured a statistically significant improvement using a rubric, but no differences between the active learning intervention group compared to the didactic learning control group for information literacy [24]. Eldredge et al. measured statistically improved scores on EBP searching as guided by rubrics, although there were no significant differences between a student peer assessment intervention group compared to the control group only engaged in hands-on searching [25]. Kloda et al. found significant overall improvement in all occupational therapy and physical therapy students' scores while at the same time no difference between either question formulation arm of their study [26]. Outside the realm of EBP, at least one other randomized controlled trial involving interventions with active learning in health professions students have similarly resulted in statistically non-significant differences between intervention and control groups. [27]

These students operated in a solely online learning environment during the Covid-19 pandemic. Perhaps an online factor such as the instructors' inability to monitor their peer learning online in all breakout rooms meant that the intervention Group students did not really perform the requested skills applications. If not, then the students would have interrupted Kolb's theoretical Experiential Learning Cycle by omitting their own Active Experimentation and the Concrete Experience phases [28]. Further, student omission of the active learning paired interactions would have curtailed the higher learning phases Bloom's theoretical phases of Apply, Analyze, Evaluate, and possibly Create [29]. Empirical studies involving health professions students tend to confirm these two theoretical aspects of active learning [30–31]. Further studies, particularly those conducted within in-person contexts, should confirm or modulate this potentially time-saving FAC rubric approach. Randomized controlled trials using the FAC rubric with different health professions students might further clarify whether the potential for the rubric, coupled to a brief explanation, will suffice for these other health professions students provided that they are similarly motivated by an impending graded event. Finally, future studies should seek to confirm that the FAC does indeed outperform PICO in positioning learners for conducting effective searches for the evidence and the critical appraisal of the identified evidence in the next two steps in the EBP process.

Medical educators regularly struggle with deciding which knowledge content and skills to include in the crowded curriculum of undergraduate medical education. This study suggests that medical students, apparently motivated by knowing that their acquiring these skills might influence their block grade, will perform well using the rubric for their EBP question formulation. Using the rubric with a brief instructional session will potentially reduce the cognitive load during classroom time for students and contribute to reducing overall congestion in the crowded curriculum. Time saved in teaching question formulation skills potentially can be reallocated to other EBP training such as searching skills.

The authors hypothesized that a 25-minute training session would result in a statistically-significant increase in scores compared to a control group receiving a 5-minute explanation of a rubric only. These results suggest that a 5-minute explanation of the rubric alone might be sufficient guidance for students motivated by an impending graded-event in using the FAC rubric to improve their scores when learning their EBP question formulation skills. Introducing students to the rubric definitely boosted all student scores. Further replication of this study at multiple sites, particularly in an in-person environment will be essential.

## Data Availability

Data associated with this article are available at the University of New Mexico Digital Repository https://digitalrepository.unm.edu/hsc_hslic/3/.

## SUPPLEMENTARY MATERIAL AVAILABILITY

The EBP Assignment for the Quantitative Medicine Block with sample student formulated questions is available at the University of New Mexico Digital Repository: https://digitalrepository.unm.edu/hslic-publications-papers/66/.
